# Supplementary material for: Electronic Effects in Cobalt Phthalocyanine Catalysts Towards Noble-Metal-Free, Photocatalytic CO2-to-CO Reduction
Source: Molecules. 2024 Oct 22;29(21):4994. doi: 10.3390/molecules29214994 (PMC11547791; doi:10.3390/molecules29214994)
Supplement: Supplementary file 1 [file molecules-29-04994-s001.zip › molecules-3261145-supplementary.pdf]

Supplementary Information (SI) for

Electronic Effects in Cobalt Phthalocyanine Catalysts Towards Noble-Metal-Free, Photocatalytic CO_2-to-CO Reduction

Fan Ma [1], Hong-Wei Lin [1], Zizi Li [1], Wen-Jing Li [1], Jia-Wei Wang [1],* Gangfeng Ouyang[1,2,3]

[1]School of Chemical Engineering and Technology, Sun Yat-sen University, Zhuhai 519082, China.;

[2]Chemistry College, Center of Advanced Analysis and Gene Sequencing, Zhengzhou University, Zhengzhou 450001, China.

[3]Guangdong Provincial Key Laboratory of Emergency Test for Dangerous Chemicals, Guangdong Institute of Analysis (China National Analytical Center Guangzhou), Guangzhou, 510070, China.

*Corresponding author: Jia-Wei Wang (wangjw89@mail.sysu.edu.cn)

Experimental Section

DFT Calculation on CoPc complexes

Cobalt phthalocyanine with varied substituents have been optimized by Perdew-Burke-Ernzerhof hybrid functional (PBE0)[1] method and def2-SVP basis set with ultrafine integration grids and Grimme's DFT-D3(BJ) empirical dispersion correction[2] by Gaussian 09 package.[3] Harmonic vibrational frequency was performed at the same level to guarantee that there is no imaginary frequency in the molecules, i.e. they locate on the minima of potential energy surface. The Hirshfeld population[4] was gained from Gaussian, while HOMO (highest occupied molecular orbital), LUMO (lowest unoccupied molecular orbital), Mayer bond order[5] and the electro density at cobalt center were yielded from Multiwfn 3.8[6] whose input files were extracted from Gaussian format checkpoint. And the snapshots of HOMO and LUMO were plotted by VMD 1.9.3[7].

Cyclic Voltammetry

Cyclic voltammetry was performed in a 25 mL gas-tight three-neck flask with a three-electrode system, where a 3 mm glassy carbon disc electrode, a platinum wire auxiliary electrode, and a platinum wire pseudo-reference electrode were placed in a 0.1 M tetrabutylammonium hexafluorophosphate ($TBAPF_6$) NMP solution (8.0 mL) or NMP/TEA (v:v = 5:1; 9.6 mL). Prior to experiments, the working electrode was polished in turn with 0.3 and 0.05 μm Al_2O_3 slurry for 3 min to obtain a mirror surface, then sonicated in water for ~60 seconds to remove debris, and finally rinsed with water. The solution was bubbled with N_2/CO_2 for 15 min prior to each experiment. Scan rate is 100 mV/s.

Photocatalytic Experiments

The photocatalytic reduction of CO_2 to CO was conducted in a 17 mL home-made reactor upon successive addition of catalyst, proton source, a CH_3CN solution containing Cu PS, corresponding diphosphine ligand and BIH, and finally TEA, under 1 atm CO_2 atmosphere at 293±2 K. Phenol was placed in a N_2-saturated Schlenk flask and molten at 70 °C prior to use. After the reaction system purged with CO_2 for 10 min, the photocatalytic reaction was initiated by irradiation under an LED light. The generated gases in the headspace were analyzed by a gas chromatography with injection of 0.10 mL gas. Gas chromatography was conducted on an Agilent 7820A gas chromatograph equipped with a thermal conductivity detector (TCD) and a 1.5 m TDX-01 packed column. The calibration was done by injecting gradient amounts of CO/H_2 into the CO_2-saturated reaction vessel and then sampling for a linear fitting of standard curve. The oven temperature was held constant at 60 °C and the inlet and detector temperature were set at 80 and 200 °C, respectively. The possible products in the solution were analyzed by ion chromatograph.

Determination of Φ for CO Production

A reported method[8] was used to determine Φ. A typical experiment employed a mixture of **CoTCPc** (0.05 mM), **CuBCP** (0.5 mM), xantphos (1.0 mM), phenol (5.0 v%), and BIH (50 mM) in 4.0 mL CH_3CN/TEA (v:v = 5:1) for evaluation. The temperature was kept at 25 °C. The light source is an LED light set-up (λ = 425 ± 5 nm, light intensity = 40 mW·cm^{-2}, irradiated area is 1.5 cm^2). The photon flux was determined to be 2.39 × 10^{-7} einstein s^{-1}.[9] Under these conditions, the light entering the reaction solution was considered to be fully absorbed by PS without scattering, suggesting the evaluated Φ is a lower limit. The 5 min of light irradiation is consistent with the total number of photons n_p = 7.18 × 10^{-5} einstein.

The Φ with BIH as the two-electron reductant was evaluated by the Equation[10] for two-electron reduction of CO_2.

$$\Phi = n(\mathrm{CO}) / n_p \quad \text{(S1)}$$

A typical Φ of 63.5% was determined by the measured n(CO) = 45.6 μmol by GC-TCD after 5 min of irradiation.

Determination of Quenching Rate (k_q)

A reported method[10] was followed to calculate the k_q, where the fluorescence intensity of photo-excited Cu(I) PS in the presence of quencher was measured and fitted to the Stern-Volmer Equation,

$$\frac{I_0}{I} = 1 + K[\mathrm{Q}] = 1 + k_q\tau_0[\mathrm{Q}] \quad \text{(S2)}$$

In this Equation, I_0 and I are the fluorescence intensity values in the absence and presence of the quencher, K is the

Stern-Volmer constant for dynamic quenching, k_q is the apparent rate of bimolecular quenching, τ_0 is the lifetime of the excited state without quencher, and [Q] is the concentration of the quencher.

Supporting Figures

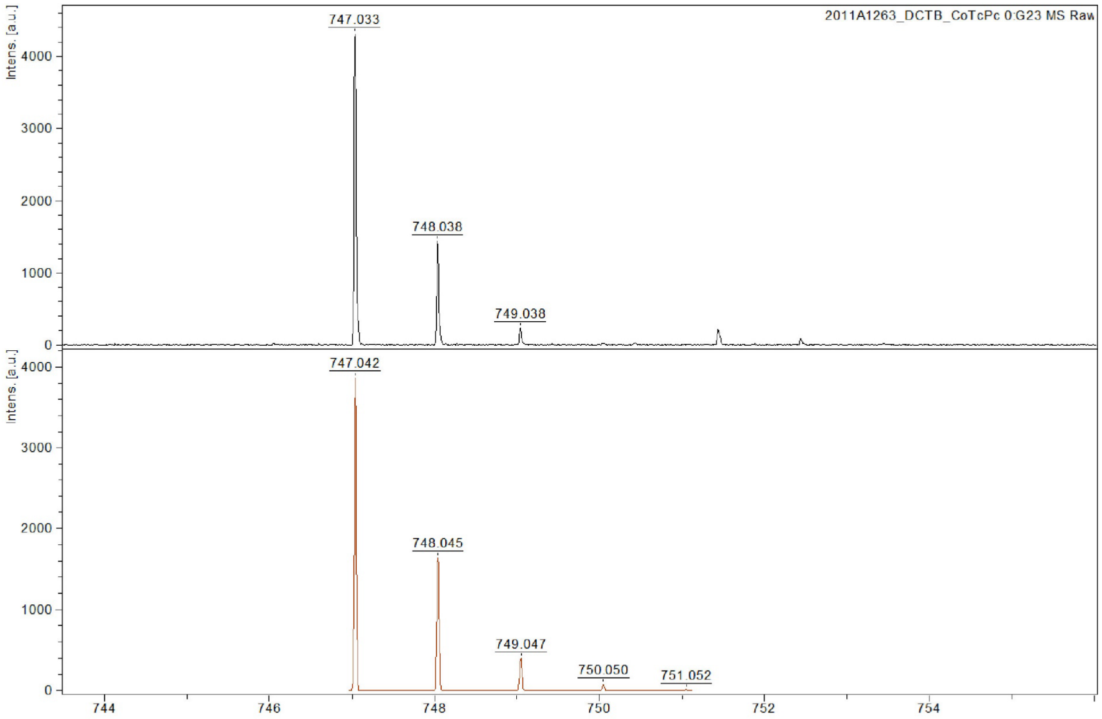

Supplementary Figure S1. MALDI-TOF HR MS spectrum of **CoTCPc** dissolved in NMP.

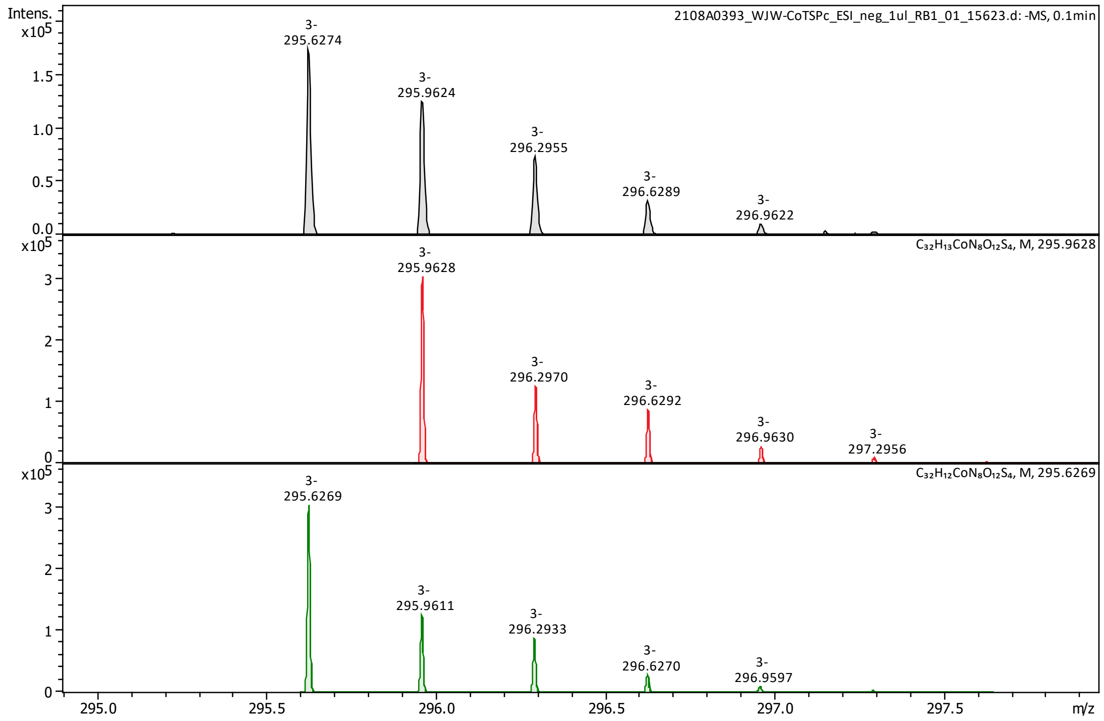

Supplementary Figure S2. MALDI-TOF HR MS spectrum of **CoTSPc** dissolved in NMP.

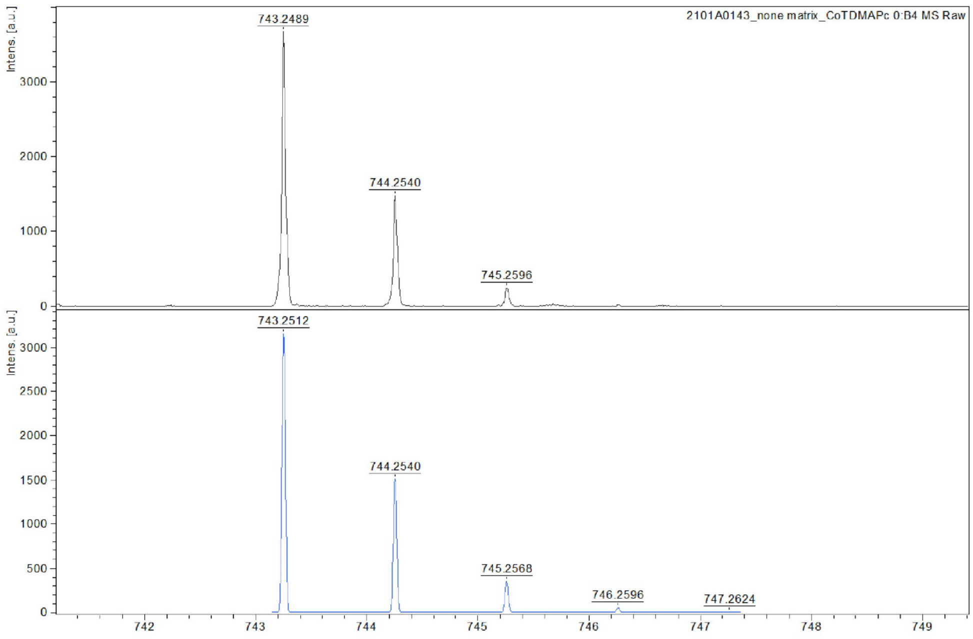

Supplementary Figure S3. MALDI-TOF HR MS spectrum of **CoTDMAPc** dissolved in NMP.

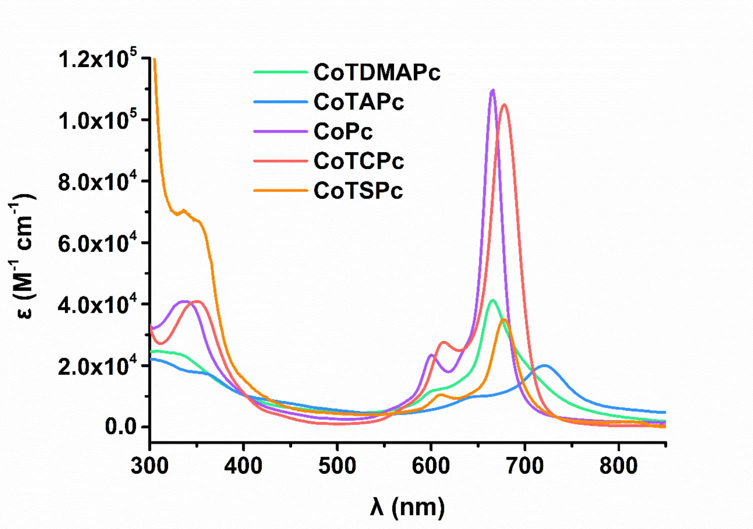

Supplementary Figure S4. UV-vis spectra of 25 μM **CoPc** derivatives in NMP.

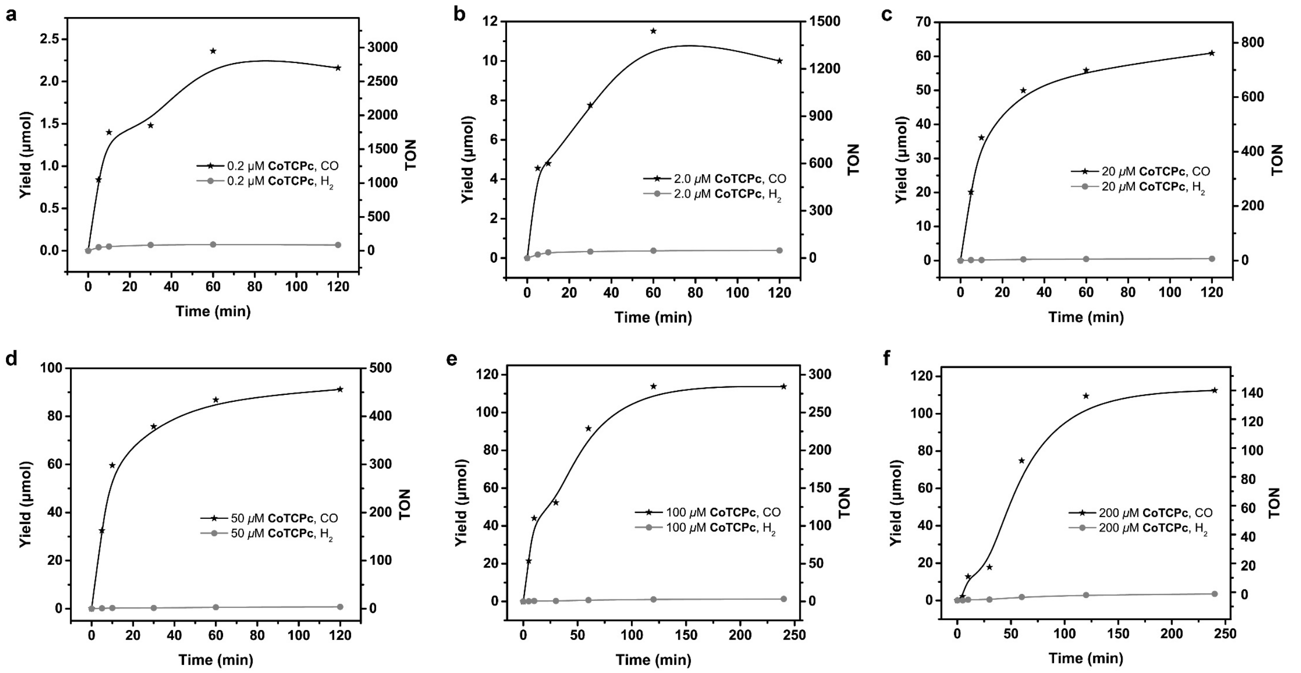

Supplementary Figure S5. Time profiles and TONs of photocatalytic CO and H_2 yields from a mixture of **CuBCP** (0.5 mM), xantphos ligand (1.0 mM), phenol (5.0 v%), and BIH (20 mM) in 4 mL CH_3CN/TEA (v:v = 5:1) within 1 h of 450-nm LED irradiation under 1 atm CO_2 with the concentration of **CoTCPc** as (a) 0.2 μM, (b) 2.0 μM, (c) 20 μM, (d) 50 μM, (e) 100 μM or (f) 200 μM.

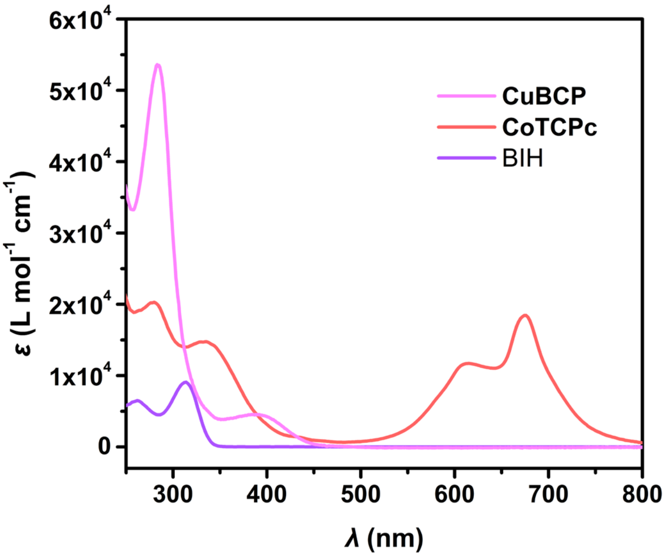

Supplementary Figure S6. UV-vis spectra of 50 μM **CuBCP** (magenta), 10 μM **CoTCPc** (red) and 10 μM BIH (volet) in CH_3CN.

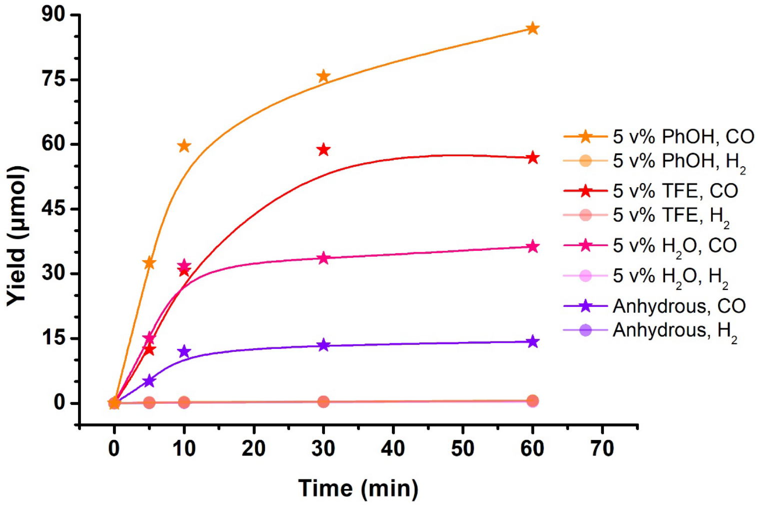

Supplementary Figure S7. Time profiles of photocatalytic CO and H_2 yields from a mixture of **CuBCP** (0.5 mM), xantphos ligand (1.0 mM), **CoTCPc** (0.05 mM), and BIH (20 mM) in 4 mL CH_3CN/TEA (v:v = 5:1) under 1 atm CO_2 without proton souce (violet) or with 5.0 v% of phenol (PhOH, orange), TFE (red) or H_2O (pink).

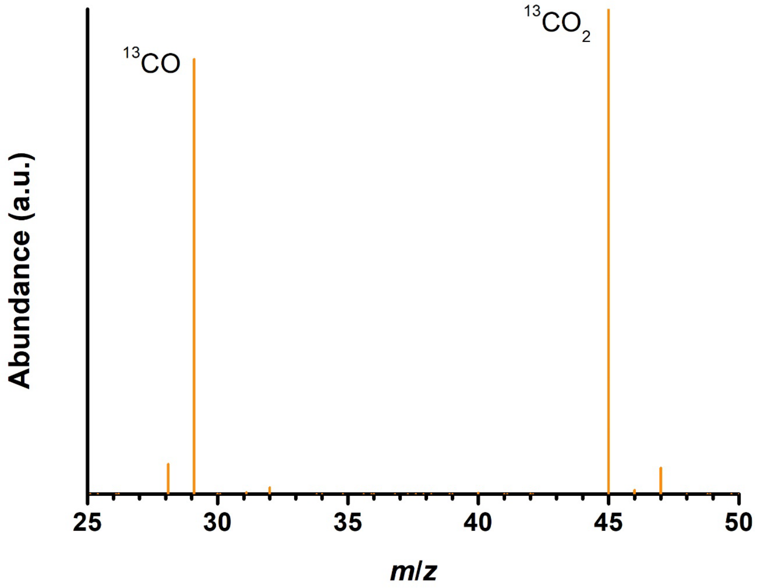

Supplementary Figure S8. Mass spectra analyses via gas chromatography on the generated gas from a mixture of **CuBCP** (0.5 mM), xantphos ligand (1.0 mM), **CoTCPc** (0.05 mM), phenol (5.0 v%), and BIH (20 mM) in 4 mL CH_3CN/TEA (v:v = 5:1) within 1 h of 450-nm LED irradiation under 1 atm CO_2.

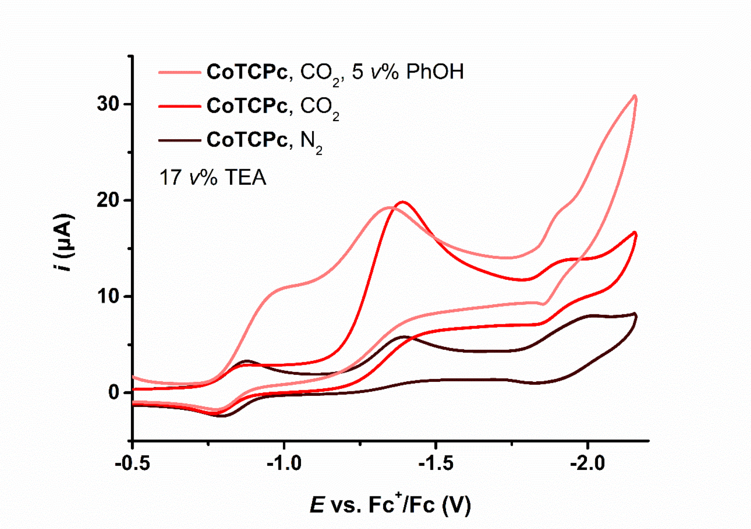

Supplementary Figure S9. CVs of 0.5 mM **CoTCPc** in 0.1 M *n*Bu$_4$NPF$_6$ NMP/TEA (v:v = 5:1) solution under N_2 (deep red), CO_2 (medium red) or CO_2 (pale red) with 5 v% phenol at a scan rate of 0.1 V s^{-1}.

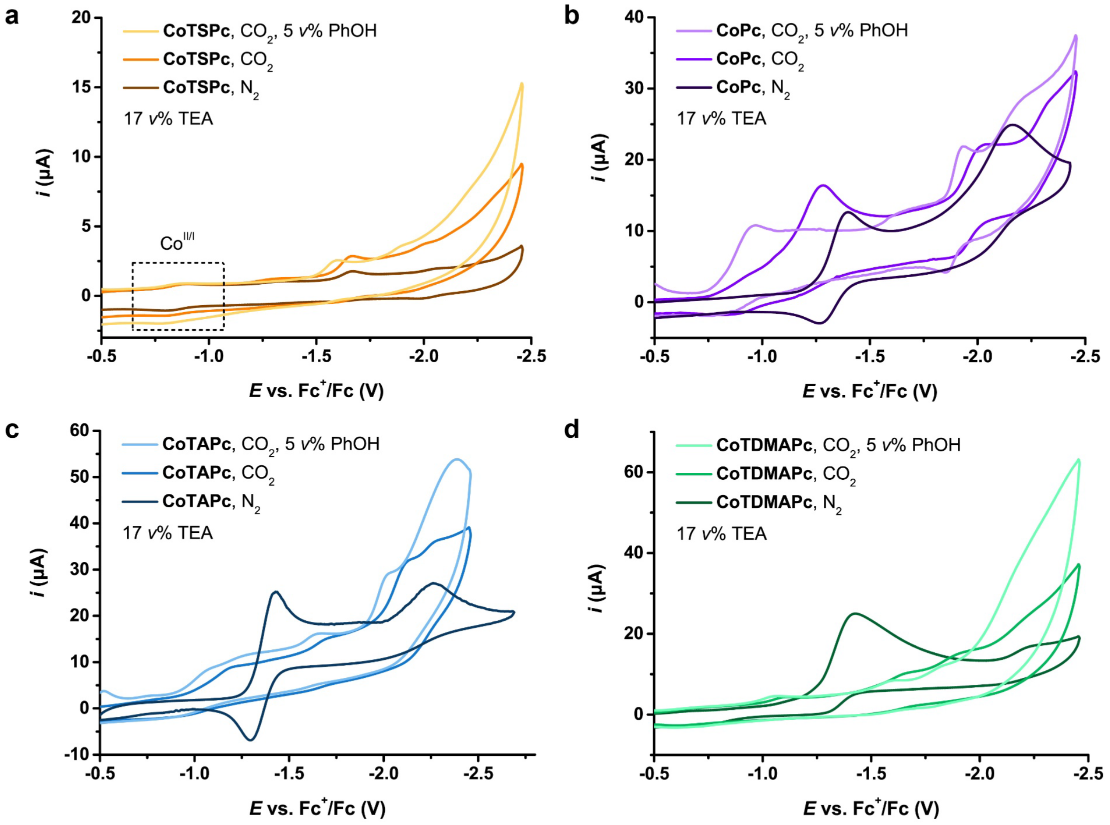

Supplementary Figure S10. CVs of (a) 0.1 mM **CoTSPc** (limited solubility), (b) 0.5 mM **CoPc**, (c) 0.5 mM **CoTAPc** or (d) 0.5 mM **CoTDMAPc** in 0.1 M *n*Bu$_4$NPF$_6$ NMP/TEA (v:v = 5:1) solution under N_2 (deep color), CO_2 (medium color) or CO_2 (pale color) with 5 v% phenol (PhOH) at a scan rate of 0.1 V s^{-1}.

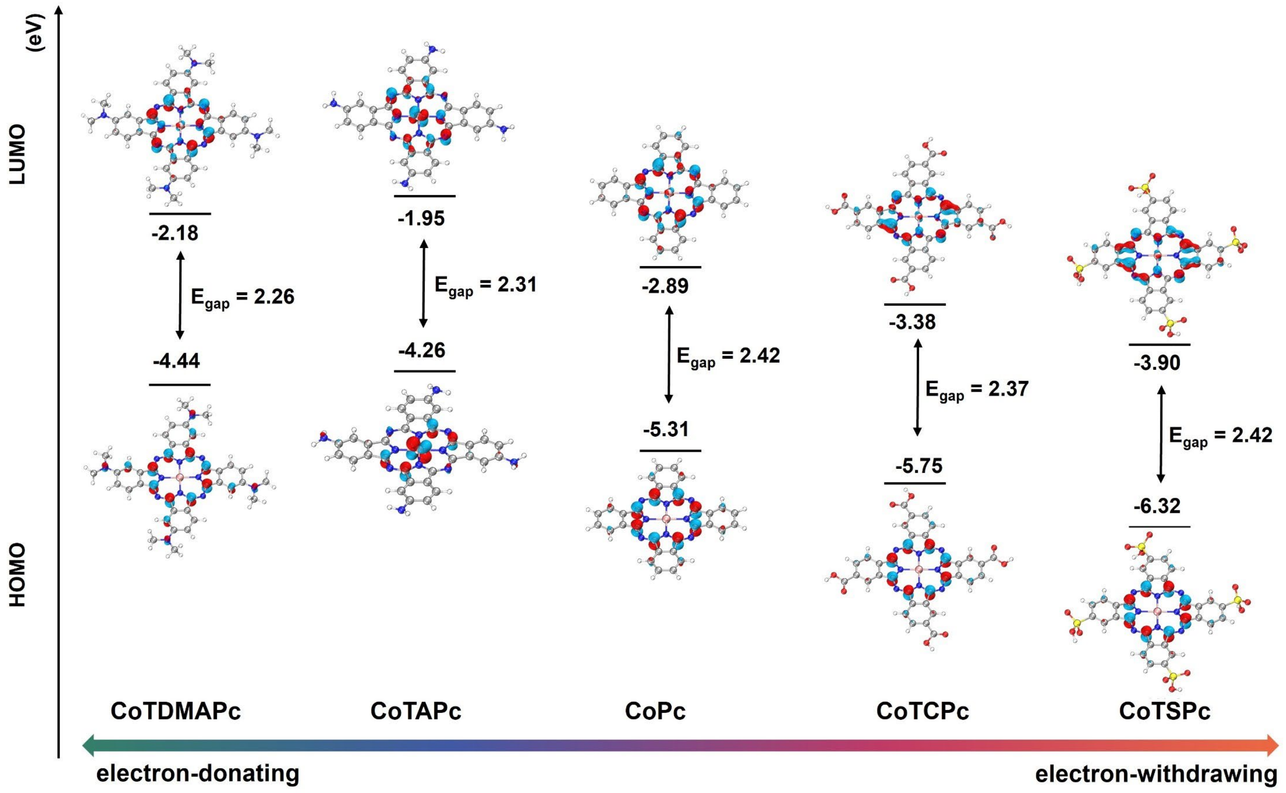

Supplementary Figure S11. The HOMO (bottom) and LUMO (top) of the studied **CoPc**-derived catalysts. The red and blue orbitals are positive and negative phases, and the isovalue of orbitals is equal to 0.05.

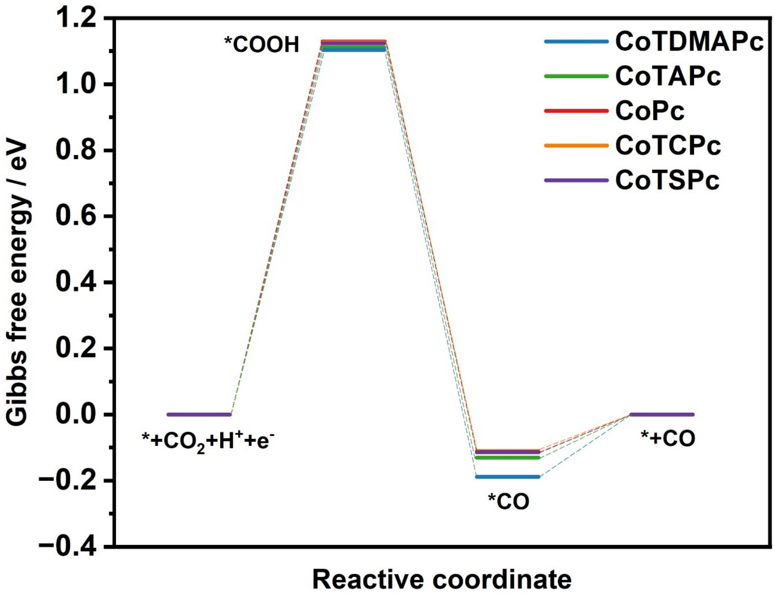

Supplementary Figure S12. The Gibbs free energy profile for the five **CoPc**-derived catalysts (eV).

Supporting Tables

Supplementary Table S1. Elemental analysis results of Cu PSs and several **CoPc** derivatives.

Complexes	Calculated formula	Calculated value (%)			Measured value (%)		
		C	H	N	C	H	N
**CuBCP**	$CuC_{65.5}H_{53}F_6N_2P_3OCl$ (**CuBCP**·$0.5CH_2Cl_2$)	66.11	4.49	2.35	66.45	4.44	2.18
**CoTCPc**	$CoC_{36}H_{28}Cl_2N_8Na_2O_{14}$ (**CoTCPc**·$6H_2O$·2NaCl)	44.46	2.90	11.52	44.42	3.02	11.86
**CoTSPc**	$CoC_{32}H_{14}Cl_2N_8NaO_{13}S_4$ (**CoTSPc**$^{4-}$·H_2O·NaCl)	36.39	1.34	10.61	36.82	1.31	10.84
**CoTDMAPc**	$C_{45}H_{47}CoI_5N_{13}Na_5O_2$ (**CoTDMAPc**·NMP·H_2O·5NaI)	33.56	2.94	11.31	33.75	2.85	11.01

Supplementary Table S2. Photocatalytic CO_2 reduction to CO by **CuBCP/CoTCPc** in the absence of certain component.*

Entry	Conditions	*n*(CO) (μmol)	*n*(H_2) (μmol)
1	No **CoTCPc**	N.D.	1.20±0.40
2	No **CuBCP**	N.D.	N.D.
3	Under N_2 instead of CO_2	N.D.	N.D.
4	No irradiation	N.D.	N.D.
5	No BIH	0.60±0.10	1.10±0.20

*Standard condition: **CuBCP** (0.5 mM), xantphos ligand (1.0 mM), **CoTCPc** (0.05 mM), phenol (5.0 v%), and BIH (20 mM) in 4 mL CH_3CN/TEA (v:v = 5:1) within 1 h of 450-nm LED irradiation under 1 atm CO_2.

Supplementary Table S3. The geometries parameters of the studied **CoPc**-derived catalysts, the Co-N and φ denote the bond lengths of Co and N atoms (Å), angles of plane distortion (°) and Mayer bond order (MBO) between Co and N atoms.

Molecules	*d*(Co-N)	φ	MBO
**CoTDMAPc**	1.9274	0.00	0.694
**CoTAPc**	1.9272	0.00	0.739
**CoPc**	1.9266	0.00	0.696
**CoTCPc**	1.9269	0.00	0.695
**CoTSPc**	1.9265	0.02	0.696

Supplementary Table S4. The HOMO-LUMO gap (eV) as well as the electron density for Co (ρ_{Co}, a.u.) atom in the studied **CoPc**-derived catalysts. (all units are a.u.)

Molecules	ρ_{Co}	HOMO-LUMO gap
**CoTDMAPc**	1.30×10^5	2.2573
**CoTAPc**	1.30×10^5	2.3122
**CoPc**	1.30×10^5	2.4223
**CoTCPc**	1.30×10^5	2.3722
**CoTSPc**	1.30×10^5	2.4173

Supplementary Table S5. The optimized structures and Gibbs free energies of *COOH and *CO intermediates from the **CoPc** families. The white, gray, blue, red, yellow and pink colors denote H, C, N, O, S and Co atoms.

Complexes	*COOH species	ΔG(*COOH) (eV)	*CO species	ΔG(*CO) (eV)
**CoTSPc**	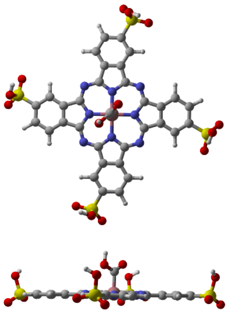	1.123817	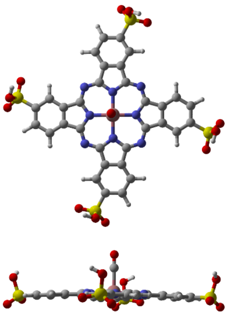	-0.11355
**CoTCPc**	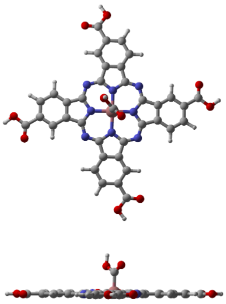	1.126483	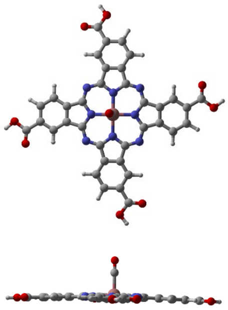	-0.11021
**CoPc**	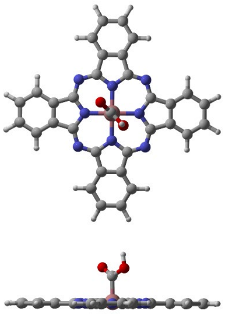	1.129477	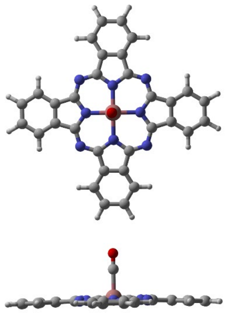	-0.11377
**CoTAPc**	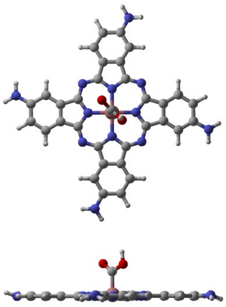	1.111136	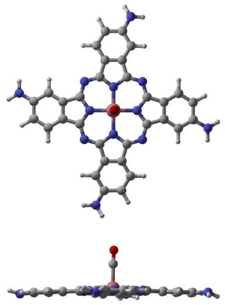	-0.13034
**CoTDMAPc**	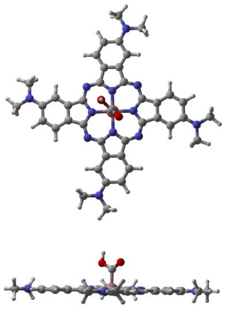	1.10349	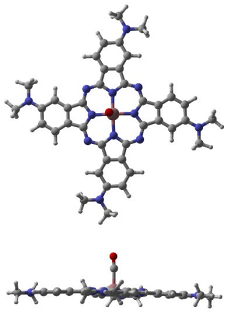	-0.18819

References

1. Adamo, C.; Barone, V., Toward reliable density functional methods without adjustable parameters: The PBE0 model. *J. Chem. Phys.* **1999,** *110* (13), 6158-6170.
2. Goerigk, L.; Grimme, S., Efficient and Accurate Double-Hybrid-Meta-GGA Density Functionals-Evaluation with the Extended GMTKN30 Database for General Main Group Thermochemistry, Kinetics, and Noncovalent Interactions. *Journal of Chemical Theory and Computation* **2011,** *7* (2), 291-309.
3. Frisch, M. J.; Trucks, G. W.; Schlegel, H. B.; Scuseria, G. E.; Robb, M. A.; Cheeseman, J. R.; Scalmani, G.; Barone, V.; Mennucci, B.; Petersson, G. A.; Nakatsuji, H.; Caricato, M.; Li, X.; Hratchian, H. P.; Izmaylov, A. F.; Bloino, J.; Zheng, G.; Sonnenberg, J. L.; Hada, M.; Ehara, M.; Toyota, K.; Fukuda, R.; Hasegawa, J.; Ishida, M.; Nakajima, T.; Honda, Y.; Kitao, O.; Nakai, H.; Vreven, T.; Montgomery, J. A., Jr.; Peralta, J. E.; Ogliaro, F.; Bearpark, M.; Heyd, J. J.; Brothers, E.; Kudin, K. N.; Staroverov, V. N.; Kobayashi, R.; Normand, J.; Raghavachari, K.; Rendell, A.; Burant, J. C.; Iyengar, S. S.; Tomasi, J.; Cossi, M.; Rega, N.; Millam, J. M.; Klene, M.; Knox, J. E.; Cross, J. B.; Bakken, V.; Adamo, C.; Jaramillo, J.; Gomperts, R.; Stratmann, R. E.; Yazyev, O. A., A. J.; Cammi, R. P., C.; Ochterski, J. W.; Martin, R. L.; Morokuma, K.; Zakrzewski, V. G.; Voth, G. A.; Salvador, P.; Dannenberg, J. J.; Dapprich, S.; Daniels, A. D.; Farkas, Ö.; Foresman, J. B.; Ortiz, J. V.; Cioslowski, J.; Fox, D. J. *Gaussian 09*, Revision C.01; Gaussian, Inc.: Wallingford CT, 2009.
4. Hirshfeld, F. L., Bonded-atom fragments for describing molecular charge densities. *Theoretica chimica acta* **1977,** *44* (2), 129-138.
5. Mayer, I., Charge, bond order and valence in the AB initio SCF theory. *Chem. Phys. Lett.* **1983,** *97* (3), 270-274.
6. Lu, T.; Chen, F., Multiwfn: a multifunctional wavefunction analyzer. *J. Comput. Chem.* **2012,** *33* (5), 580-92.
7. Humphrey, W.; Dalke, A.; Schulten, K., VMD: visual molecular dynamics. *Journal of molecular graphics* **1996,** *14* (1), 33-38.
8. Li, H.; Li, F.; Zhang, B.; Zhou, X.; Yu, F.; Sun, L., Visible light-driven water oxidation promoted by host-guest interaction between photosensitizer and catalyst with a high quantum efficiency. *J. Am. Chem. Soc.* **2015,** *137* (13), 4332-4335.
9. Kuhn, H.; Braslavsky, S.; Schmidt, R., Chemical actinometry (IUPAC technical report). *Pure Appl. Chem.* **2004,** *76* (12), 2105-2146.
10. Thoi, V. S.; Kornienko, N.; Margarit, C. G.; Yang, P.; Chang, C. J., Visible-light photoredox catalysis: selective reduction of carbon dioxide to carbon monoxide by a nickel N-heterocyclic carbene-isoquinoline complex. *J. Am. Chem. Soc.* **2013,** *135* (38), 14413-14424.

Supplementary File S1: DFT calculation data on **CoPc** and its derivatives

The Hirshfeld atomic charges (e) of studied molecules. The number denotes the atomic labels.

Molecules	Atoms	Charge	Atoms	Charge
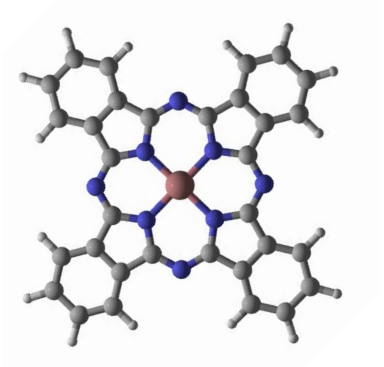	C1	-0.0282	C29	-0.0223
	C2	-0.0352	C30	-0.0282
	C3	-0.0351	C31	-0.0352
	C4	-0.0282	C32	-0.0352
	C5	-0.0223	C33	-0.0282
	C6	0.1020	C34	-0.0223
**CoPc**	N7	-0.1533	C35	0.1019
	C8	0.1020	N36	-0.1178
	C9	-0.0223	N37	-0.1532
	C10	-0.0282	C38	0.1020
	C11	-0.0352	N39	-0.1178
	C12	-0.0351	C40	-0.0223
	C13	-0.0282	H41	0.0493
	C14	-0.0223	H42	0.0446
	C15	0.1020	H43	0.0446
	N16	-0.1179	H44	0.0493
	N17	-0.1531	H45	0.0492
	C18	0.1019	H46	0.0446
	C19	-0.0223	H47	0.0446
	C20	-0.0282	H48	0.0492
	C21	-0.0352	H49	0.0492
	C22	-0.0352	H50	0.0446
	C23	-0.0282	H51	0.0446
	C24	-0.0223	H52	0.0492
	C25	0.1019	H53	0.0492
	N26	-0.1178	H54	0.0446

	N27	-0.1532	H55	0.0446
	C28	0.1019	H56	0.0492
			Co57	0.2038
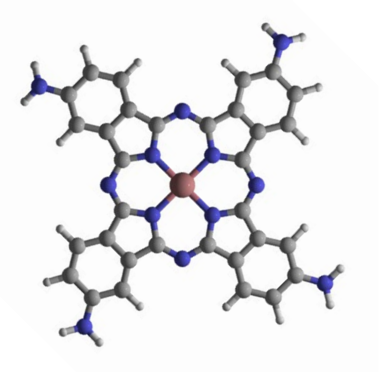	C1	-0.0294	C34	-0.0223
	C2	-0.0659	C35	0.0982
	C3	0.0510	N36	-0.1220
	C4	-0.0634	N37	-0.1607
	C5	-0.0223	C38	0.0974
	C6	0.0982	N39	-0.1220
**CoTAPc**	N7	-0.1607	C40	-0.0421
	C8	0.0974	H41	0.0472
	C9	-0.0421	H42	0.0358
	C10	-0.0294	H43	0.0405
	C11	-0.0659	H44	0.0472
	C12	0.0510	H45	0.0358
	C13	-0.0634	H46	0.0405
	C14	-0.0223	H47	0.0472
	C15	0.0982	H48	0.0358
	N16	-0.1220	H49	0.0405
	N17	-0.1607	H50	0.0472
	C18	0.0974	H51	0.0358
	C19	-0.0421	H52	0.0405
	C20	-0.0294	Co53	0.1971
	C21	-0.0659	N54	-0.1614
	C22	0.0510	H55	0.1232
	C23	-0.0634	H56	0.1247
	C24	-0.0223	N57	-0.1614
	C25	0.0982	H58	-0.0294
	N26	-0.1220	H59	-0.0659

	N27	-0.1607	N60	0.0510
	C28	0.0974	H61	-0.0634
	C29	-0.0421	H62	-0.0223
	C30	-0.0294	N63	0.0982
	C31	-0.0659	H64	-0.1607
	C32	0.0510	H65	0.0974
	C33	-0.0634		
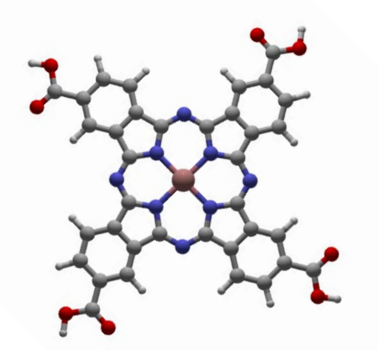	C1	-0.0236	C35	0.1056
	C2	-0.0255	N36	-0.1151
	C3	-0.0168	N37	-0.1482
	C4	-0.0110	C38	0.1046
	C5	-0.0200	N39	-0.1151
	C6	0.1056	C40	-0.0134
**CoTCPc**	N7	-0.1482	H41	0.0545
	C8	0.1046	H42	0.0478
	C9	-0.0134	H43	0.0557
	C10	-0.0236	H44	0.0545
	C11	-0.0255	H45	0.0478
	C12	-0.0168	H46	0.0557
	C13	-0.0110	H47	0.0545
	C14	-0.0200	H48	0.0478
	C15	0.1056	H49	0.0557
	N16	-0.1151	H50	0.0545
	N17	-0.1482	H51	0.0478
	C18	0.1046	H52	0.0557
	C19	-0.0134	Co53	0.2084
	C20	-0.0236	C54	0.2095
	C21	-0.0255	O55	-0.2781
	C22	-0.0168	O56	-0.1666

	C23	-0.0110	H57	0.1885
	C24	-0.0200	C58	0.2095
	C25	0.1056	O59	-0.2781
	N26	-0.1151	O60	-0.1666
	N27	-0.1482	H61	0.1885
	C28	0.1046	C62	0.2095
	C29	-0.0134	O63	-0.2781
	C30	-0.0236	O64	-0.1666
	C31	-0.0255	H65	0.1885
	C32	-0.0168	C66	0.2095
	C33	-0.0110	O67	-0.2781
	C34	-0.0200	O68	-0.1666
			H69	0.1885
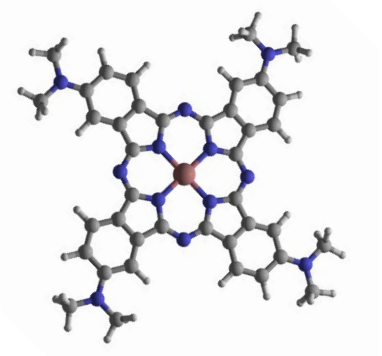	C1	-0.0317	H46	0.0373
	C2	-0.0683	H47	0.0457
	C3	0.0460	H48	0.0330
	C4	-0.0652	H49	0.0373
	C5	-0.0241	H50	0.0457
	C6	0.0977	H51	0.0330
**CoTDMAPc**	N7	-0.1616	H52	0.0373
	C8	0.0966	Co53	0.1959
	C9	-0.0436	N54	-0.0419
	C10	-0.0317	N55	-0.0419
	C11	-0.0683	N56	-0.0419
	C12	0.0460	N57	-0.0419
	C13	-0.0652	C58	-0.0316
	C14	-0.0241	H59	0.0420
	C15	0.0977	H60	0.0327
	N16	-0.1224	H61	0.0327

N17	-0.1616	C62	-0.0315
C18	0.0966	H63	0.0340
C19	-0.0436	H64	0.0413
C20	-0.0317	H65	0.0340
C21	-0.0683	C66	-0.0316
C22	0.0460	H67	0.0420
C23	-0.0652	H68	0.0327
C24	-0.0241	H69	0.0326
C25	0.0977	C70	-0.0317
N26	-0.1224	H71	-0.0683
N27	-0.1616	H72	0.0460
C28	0.0966	H73	-0.0652
C29	-0.0436	C74	-0.0241
C30	-0.0317	H75	0.0977
C31	-0.0683	H76	-0.1616
C32	0.0460	H77	0.0966
C33	-0.0652	C78	-0.0436
C34	-0.0241	H79	-0.0317
C35	0.0977	H80	-0.0683
N36	-0.1224	H81	0.0460
N37	-0.1616	C82	-0.0652
C38	0.0966	H83	-0.0241
N39	-0.1224	H84	0.0977
C40	-0.0436	H85	-0.1224
H41	0.0457	C86	-0.1616
H42	0.0330	H87	0.0966
H43	0.0373	H88	-0.0436
H44	0.0457	H89	-0.0317
H45	0.0330		

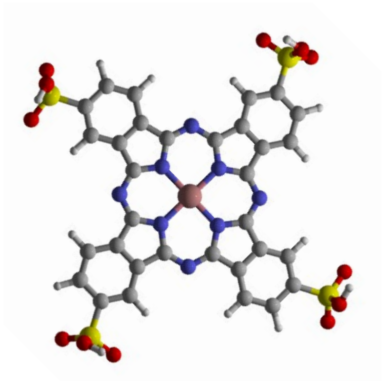

CoTSPc

C1	-0.0146	N37	-0.1458
C2	-0.0174	C38	0.1068
C3	-0.0239	N39	-0.1137
C4	-0.0172	C40	-0.0110
C5	-0.0164	H41	0.0592
C6	0.1070	H42	0.0596
N7	-0.1458	H43	0.0573
C8	0.1068	H44	0.0592
C9	-0.0110	H45	0.0596
C10	-0.0146	H46	0.0573
C11	-0.0174	H47	0.0592
C12	-0.0239	H48	0.0596
C13	-0.0172	H49	0.0573
C14	-0.0164	H50	0.0592
C15	0.1070	H51	0.0596
N16	-0.1137	H52	0.0573
N17	-0.1458	Co53	0.2122
C18	0.1068	S54	0.5453
C19	-0.0110	O55	-0.3119
C20	-0.0146	O56	-0.2953
C21	-0.0174	S57	0.5453
C22	-0.0239	O58	-0.3119
C23	-0.0172	O59	-0.2953
C24	-0.0164	S60	0.5453
C25	0.1070	O61	-0.3119
N26	-0.1137	O62	-0.2953
N27	-0.1458	S63	0.5453
C28	0.1068	O64	-0.3119
C29	-0.0110	O65	-0.2953

C30	-0.0146	O66	-0.2095
C31	-0.0174	H67	0.1886
C32	-0.0239	O68	-0.2095
C33	-0.0172	H69	0.1886
C34	-0.0164	O70	-0.0146
C35	0.1070	H71	-0.0174
N36	-0.1137	O72	-0.0239
		H73	-0.0172
